# Self-Powered Portable Electronic Reader for Point-of-Care Amperometric Measurements

**DOI:** 10.3390/s19173715

**Published:** 2019-08-27

**Authors:** Yaiza Montes-Cebrián, Albert Álvarez-Carulla, Jordi Colomer-Farrarons, Manel Puig-Vidal, Pere Ll. Miribel-Català

**Affiliations:** Department of Electronics and Biomedical Engineering, Faculty of Physics, University of Barcelona, Martí i Franquès 1, 08028 Barcelona, Spain

**Keywords:** self-powered, point-of-care diagnostics, low power electronics, smart electronics, portable, amperometric measurements

## Abstract

In this work, we present a self-powered electronic reader (e-reader) for point-of-care diagnostics based on the use of a fuel cell (FC) which works as a power source and as a sensor. The self-powered e-reader extracts the energy from the FC to supply the electronic components concomitantly, while performing the detection of the fuel concentration. The designed electronics rely on straightforward standards for low power consumption, resulting in a robust and low power device without needing an external power source. Besides, the custom electronic instrumentation platform can process and display fuel concentration without requiring any type of laboratory equipment. In this study, we present the electronics system in detail and describe all modules that make up the system. Furthermore, we validate the device’s operation with different emulated FCs and sensors presented in the literature. The e-reader can be adjusted to numerous current ranges up to 3 mA, with a 13 nA resolution and an uncertainty of 1.8%. Besides, it only consumes 900 µW in the low power mode of operation, and it can operate with a minimum voltage of 330 mV. This concept can be extended to a wide range of fields, from biomedical to environmental applications.

## 1. Introduction

The use of a fuel cell (FC) to replace conventional batteries in portable systems is of great interest [1,2,3]. FCs open up new opportunities, mainly in the field of consumer and industrial portable devices. There is a particular interest in the field of diagnostic tools based on biofuel cells, notably in the frame of point-of-care (POC) devices and biosensors for environmental monitoring [4]. In such systems, a big challenge is to implement self-powered sensing devices [5].

The general architecture of such devices is defined mainly by three modules. On the one hand the sensor and the power source, which can be a conventional battery, sensor or a FC, and on the other hand, the electronic reader (e-reader), which is the center of interest in the present work. 

Particularly, portable and wearable devices are of interest due to their applications in health monitoring, diagnostics, prevention, among others [6,7]. In most cases, these systems use batteries as power sources. Generally, developed countries have a good electricity grid and batteries are inexpensive. The problem arises if we focus on the developing world, where they are in most need of these kind of portable diagnostic devices [8]. Furthermore, batteries are limited by their reduced lifetime, negative environmental impact, large size, and weight. Different approaches are looking for the best way to power a POC solution [9]. An example is paper-based assays (PBAs), wherein sample manipulation, pre-treatment, and electronics are not required [5] since they usually use colorimetric systems to read the results [10]. Promising alternative solutions are emerging whereby the energy from the environment is used by energy harvesters to produce power, which can be used to supply portable and wearable devices. There are many approaches to harvest energy based on biofuel cells (BFCs) [11,12], thermo-electric generators (TEG) [13], piezoelectric generators (PZT) [14], among others.

FCs are electrochemical devices that harvest electrical energy from chemical reactions. They can work as a power source and as a sensor because they produce electricity for as long as there is available fuel. Their output current is directly correlated with the concentration of fuel. Two sub-categories of FCs are enzymatic fuel cells (EFCs) and microbial fuel cells (MFCs) [15,16]. Both have similar operational principles for energy production. EFCs produce electrical power using enzymes as a catalyst to oxidize their fuel, while MFCs use bacteria as the catalysts to oxidize organic and inorganic matter.

So far, it is only possible to power low-consumption microelectronic systems with these kinds of FCs since their power density output ranges from μW up to mW. Due to the fact that EFCs have higher power density and a more compact size, they are suitable to power portable and wearable electronic solutions [11,16]. Nevertheless, MFCs are an eco-friendly way of producing electrical energy from waste, since the electricity can come from waste material. This waste material becomes cleaner after the break-down process and can be directly discharged to the environment [17]. Moreover, MFCs may be employed as long-term power generating systems for online environment quality monitoring [18].

An ideal scenario is based on an FC which uses the same sample to power electronics and sense the concentration. However, the FC can be replaced by other power sources like standard batteries, printed batteries, energy-harvesting resources or a combination of such solutions. For instance, a possible scenario is a FC that supplies the electronics and a sensor that measures the analyte concentration. The sensor can be an amperometric biosensor to monitor oxygen and catechol [19] or glucose [20]. Furthermore, there are examples of amperometric systems based on the detection of the inhibition of glucose oxidase enzyme [21] or horseradish peroxidase activity [22]. Another interesting case is the air/water quality monitoring using FCs as a power source along with amperometric or potentiometric sensors [23,24]. In [25], a degradable battery was proposed fabricated using organic materials. It was able to operate up to 100 min with an adaptable output voltage from 1.5 to 3.0 V. The battery could be combined with amperometric biosensors to power a commercial water quality POC and detect heavy metals. Moreover, the device can be powered from polluted air generated during the treatment of wastewater [26], or in space missions [27,28] where FCs generate electrical power from hydrogen and oxygen.

Systems based on commercial off-the-shield (COTS) integrated circuits (IC) are an affordable way to develop biomedical biosensing systems. Some examples of COTS-based systems have been reported in the literature. In this context, Baingane et al. developed a self-powered biosensor system for real-time sensing of lactic acid [29]. The system was comprised of an enzymatic biofuel cell and a simple capacitor circuit operating as a transducer. The fuel cell-based sensor generated an electrical power proportional to the lactic acid concentration, which was deduced by measuring the charging/discharging frequency of the capacitor circuit. The system did not rely on external power sources, although it required external equipment, like an oscilloscope, to monitor the capacitor frequency. Some works proposed the use of FC to monitor lactate in sweat. An example is a non-invasive system designed by Garcia et al. [30]. The device was composed of an enzymatic sensor, a biological FC as a power source and an electronic system. The electronic circuit consisted of an energy harvester, which boosted the FC voltage, and a potentiostat that performed a chronoamperometric detection. However, the system needed an external multimeter to perform the readout. In this context, an electronic-skin-based biofuel cell was developed by Bandodkar et al. [31]. The device harvested lactate present in human sweat to power a light-emitting diode and a bluetooth low energy radio. Other authors also proposed wireless systems [32], in which an operation using an organic biofuel cell was demonstrated. The full-custom circuit was able to operate with a voltage supply of 0.23 V. Besides, the authors designed and validated a receiver and off-chip inductor to transmit the data. 

In the particular case of glucometers, recent innovative concepts have been proposed, which are capable of generating electrical power from the biochemical energy stored in glucose [33,34]. An example is the self-powered fuel cell-based system for glucose monitoring developed by Fischer et al. [35]. The low-cost device operated with a single drop of 20 µL and lacked external power sources with the capacity to generate rapid results, although it required a digital multi-meter for signal readout. These monitoring approaches have a simple electronic system capable of extracting the glucose level from the sample. However, most of them are subjective because they cannot store and process the information [12,36].

The motivation of the batteryless e-reader derives from the needs of POC devices in different scenarios [8,37,38]. The e-reader is an equipment with a quantitative output reading that seeks to achieve the requirements defined by the World Health Organization with the ASSURED criteria (Affordable, Sensitive, Specific, User-friendly, Rapid and Robust, Equipment-free, and Deliverable to users [39]). 

In this work, we present in detail, the electronic modules that build up an e-reader based on the use of FC, following two approaches: Using the FC as a power element or as a dual power/sensor element. This power/sensor element is an external item that is inserted into the e-reader in which the same sample is used for powering and detecting. The e-reader was previously validated with a custom disposable test strip composed of a glucose battery, which supplied the device, and a glucose FC, which monitored the glucose concentration [20]. However, the e-reader can be adapted to operate in other scenarios, with another kind of FC, as power sources or biosensors. The e-reader was validated with a FC of simulated body fluid with an ionic composition similar to human blood plasma enriched with ethanol [40]. This FC has an open circuit voltage (OCV) of 930 mV with a maximum power density close of 1.237 mW·cm^−2^. Another example of application is the urine/Cr(VI) FC with an OCV of 1.3 V and a power of 340 μW·cm^−2^ [41]. In both cases, the e-reader could be supplied from the energy extracted by the fuel. At the same time, the concentration could be obtained from the current provided by the FC. Also, it is possible to perform a combination with another power source, like a direct methanol fuel cell (DMFC) for portable applications [42,43], with a specific amperometric sensor [44,45]. We describe in detail the conception, design, implementation, and characterization of the electronic modules that build-up the electronic reader based on a FC as a power source and a direct reading interface for a FC or an amperometric biosensor. Moreover, the operation of the reader is validated for different scenarios by emulating different FCs and sensors.

## 2. Materials and Methods

### 2.1. Architecture of the Self-Powered Measurement 

As it has been pointed out before, the e-reader is conceived to operate with a FC as a power source and as a sensor, taking a direct amperometric reading of the FC current. Also, it is possible to use any amperometric biosensor based on a two-electrode configuration, although configuration can be easily extended to a three-electrode configuration. Using this technique, when a potential is applied to the counter electrode (CE), a current is produced as a consequence of the reaction at the working electrode (WE) surface.

The general architecture of the system is defined mainly by two modules: The power and sensor unit and the e-reader unit. The power source can be based on paper, as was presented in [20], where the device was validated with different glucose concentrations. 

**Figure 1:**
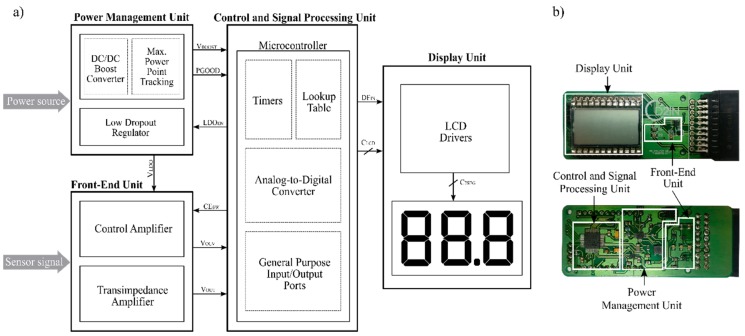
(**a**) Block diagram of the e-reader, and (**b**) picture of the e-reader, the four principal modules are labeled, derived from [20].

The architecture of the e-reader is divided into four modules (Figure 1): A power management unit (PMU), a front-end unit (FEU), a control and signal processing unit (CSPU) and a display unit (DU). The PMU extracts the power from the power source and manages it to generate the control signals and voltage supplies. All the while, the FEU picks up the measurement signal, which is proportional to the concentration of the agent to be detected. Lastly, the output signal is converted and processed by the CSPU and it is sent to the DU, where the measurement result is shown.

#### 2.1.1. Power Management Unit

**Figure 2:**
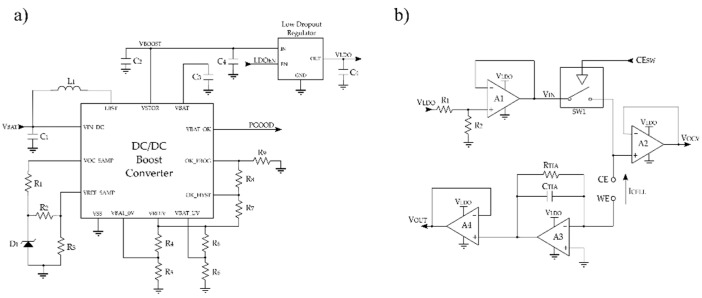
Circuits that compose the (**a**) power management unit, and (**b**) front-end unit circuit.

The PMU extracts the energy from the power source and generates a regulated output voltage to supply the electronic modules that compose the e-reader. It is comprised of a DC/DC boost converter in cascade with a low drop out (LDO) linear regulator (Figure 2a). The DC/DC boost converter steps up the voltage provided by the power source to 3.0 V (V_BOOST_) to supply both the CSPU and the DU.

The LDO provides a 1.8 V (V_LDO_) regulated voltage with high noise rejection, which is used to supply the analog components that compose the FEU. This electronic module provides a stable and regulated voltage avoiding the switching noise of the boost converter and the voltage variations of the power supply. 

Moreover, a maximum power point tracking (MPPT) module is implemented in the PMU. It guarantees an efficient energy extraction from the power source. This module can cope with a punctual high-power demand, such as the system’s start-up. For that purpose, the MPPT module adapts the input impedance of the DC/DC boost converter and changes the operating point of the power source to the voltage at which the power source provides the maximum power. This module allows managing the energy intelligently through the microcontroller. The DC/DC boost converter incorporates the PGOOD (power good) signal (Figure 2a). This signal is at a low level by default but, when the converter (V_BOOST_) reaches 2.9 V, this signal goes high state. However, if the voltage falls below 2.4 V, it returns to a low level. In this way, the microcontroller, which operates at 1.8 V, can enable/disable the LDO through the LDO_EN_ signal. Moreover, as it will be explained in the next sections, the microcontroller has different ultra-low-power modes (LPMs) in order to adapt the system operation to the available energy.

The PMU module is based on the BQ25504 and the LP5910 LDO (Texas Instruments; Dallas, TX, USA). Both devices have a very marked low power character with a typical quiescent current of 330 nA for the first one and 12 µA for the second one in shutdown mode. The total consumption of this PMU module is 21 µA. 

#### 2.1.2. Front-End Unit

The current measurement was performed by a potentiostat amplifier (Figure 2b). The main tasks of this module are: (a) To apply a stable voltage difference between the electrodes of the electrochemical cell, and (b) to readout and process the output signal.

In order to control the potential applied to the cell, an operational amplifier (Op-Amp) is used as a control amplifier (A1). A1 provides the optimal bias voltage to the sensor (V_IN_), which is the voltage that enables the best sensor’s performance. The e-reader is designed to adjust the voltage V_IN_, adapting the system to a wide range of applications. The voltage V_IN_ is generated through the low-dropout regulator (V_LDO_), and a voltage divider resistor network (R_1_ and R_2_). Because the resistor network modifies the measure, a unity gain buffer amplifier (A1) is introduced between the network and the sensor to isolate both parts.

As stated above, it is possible that the power source needs some time to reach the voltage to supply the electronic components (1.8 V). Therefore, when the PGOOD signal is at a high-level voltage, the microcontroller sends a high-level signal (LDO_EN_) to turn on the LDO ensuring the e-reader measures when enough energy is harvested to start the application operation.

For the best performance, the open circuit voltage (V_OCV_) applied to the counter electrode (CE) is monitored by an analog to digital converter (ADC) pin of the microcontroller. The ADC controls the V_OCV_ applied to the sensor and when the sample is available, it starts the measurement turning on the analog switch (SW1) through the CE_SW_ signal, depicted in Figure 2b. Since the microcontroller’s port impedance can affect the measurement, a unity gain buffer amplifier (A2) is connected between the CE and the microcontroller. 

The Transimpedance Amplifier circuit (TIA) (A3), connected to the working electrode (WE), provides the output voltage (V_OUT_) that is proportional to the cell current (I_CELL_). It translates the current signal into a voltage signal by means of transimpedance gain resistor (R_TIA_). The expression that relates both magnitudes is shown in Equation (1). In this configuration, the working electrode is kept to the virtual ground by the TIA. As before, a unity gain buffer amplifier (A4) is connected between the TIA and the microcontroller so as not to affect the measurement value.

VOUT = RTIA · ICELL(1)

The current generated by the sensor is amplified by the TIA and translated to an output voltage through the R_TIA_. Ideally, this current flows through R_TIA_, but in fact, the op-amp takes some of this current (called input bias current). A similar error is derived from the input offset voltage, which is caused by a mismatch in the input terminals of the op-amp. It specifies the voltage across the terminals that must be applied in order to get an output voltage of zero. This input bias current and input offset voltage results in an error voltage at the output and limits the dynamic range of the FEU circuit. Therefore, it is important to select an op-amp with low input bias current and low input offset voltage, to achieve the required dynamic range and overall accuracy. The selected op-amp in the FEU is the LPV521 (Texas Instruments; Dallas, TX, USA). It is a nano-power amplifier which is able to operate from 1.6 V up to 5.5 V with typically 345 nA of supply current. It also presents a very low offset voltage (0.1 mV, typically at 1.8 V) and an ultra-low bias current (0.01 pA, typically at 1.8 V). The maximum output current is 3 mA, which is the maximum current that the e-reader can read.

The analog switch used is the SN74AUC1G66 (Texas Instruments; Dallas, TX, USA). It presents a low power character since it is able to operate at 0.8 V to 2.7 V with only 10 µA of typical quiescent current. In addition, the design is made up of accurate resistors with 1% tolerance. The total consumption of the front-end unit circuit is 7.5 µA.

#### 2.1.3. Control and Signal Processing Unit

This unit is based on a low power microcontroller, which has three main tasks: a) Generate the signals that control the modules of the system; b) process the data and; c) show it on the display.

**Figure 3:**
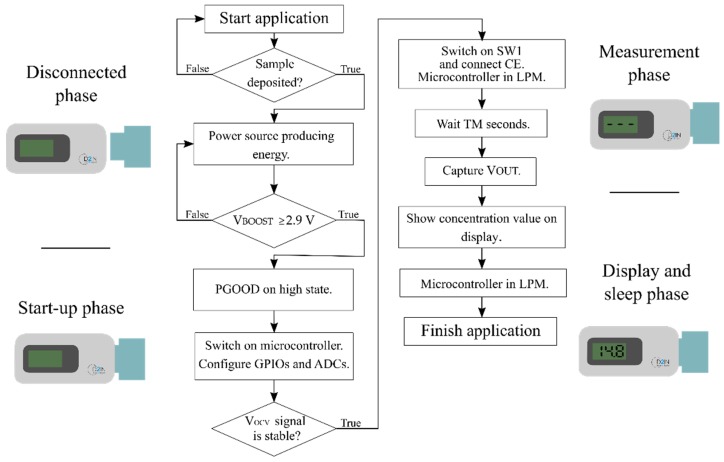
Diagram of the application’s flow.

Figure 3 shows the flow diagram of the application. Initially, the device is switched off, waiting for the introduction of the power and sensor module (“Disconnected phase”) into the reader. As soon as it is inserted and the sample deposited, the “start-up phase” begins.

Thereafter, the FC-based power source starts to deliver energy to the system, and when the output voltage of the boost converter (V_BOOST_) reaches 1.8 V (the minimum voltage to turn on the microcontroller) the microcontroller is switched on. It configures and initializes general-purpose input/output (GPIO) pins as well as the ADCs to monitor the sample and control the power delivered by the FC. 

The PGOOD signal (Figure 2b) monitors the state of the regulated voltage supply (V_BOOST_). It ensures that the e-reader performs the measurement when enough energy is harvested to start the application operation. In order to minimize power consumption, meanwhile there is not enough energy to start the measurement (PGOOD signal in a low state), the LDO remains disabled and the microcontroller stays in the low power mode (LPM). When the boost converter (V_BOOST_) reaches 2.9 V, the PGOOD changes to the high state. Afterwards, an interruption event wakes up the microcontroller from LPM, and it enables the LDO, which supplies the FEU’s components. 

Then, the ADC pin (V_OCV_) begins to monitor the state of the sensor’s voltage. The “measurement phase” starts as soon as the signal provided by the sensor is stable.

Afterwards, the microcontroller turns on the analog switch (SW1), which connects the CE, and applies a DC bias voltage (V_IN_) to the sensor as is depicted in Figure 2b. Then, a timer starts to count until the moment in which the measurement takes place, TM. TM is the time that elapses between the CE’s connection and the measurement takes place, fixed for each particular biosensor. During this time, the microcontroller remains in LPM. The criteria for determining the TM time is described in Section 3.2.

After the TM, the microcontroller exits the LPM state and the ADC captures the measurement signal (V_OUT_), which is proportional to the current provided by the sensor (I_CELL_). I_CELL_ can be obtained by Equation (1), as the input bias voltage (V_IN_), the TIA resistor (R_TIA_) and the output voltage (V_OUT_) are known.

In most situations, the relationship between the magnitude to measure and the voltage obtained is not linear. For this reason, we implemented a lookup table (LUT) [46,47,48] in the microcontroller to translate the data obtained to a current or concentration. A LUT is an array of data that allows linking input values to output values, replacing the runtime computation with a simple array indexing operation. It maps inputs to an output value by looking up or interpolating in a defined table of values.

Later, following Figure 3, the “display and sleep phase” takes place. In this phase, the microcontroller manages the drivers that control the display and shows the concentration value on the display. Finally, the microcontroller returns back to the LPM and remains in this state permanently.

The CSPU module is based on the MSP430FR5969 microcontroller (Texas Instruments; Dallas, Texas, USA). This microcontroller has been selected because of its good characteristics in terms of voltage, quiescent current and operating power consumption. It has a supply voltage that ranges from 1.8 V up to 3.6 V with one operating active mode and seven software-selectable low power modes. Furthermore, it consumes only 0.4 µA/MHz in standby mode and 100 µA/MHz in active mode. The microcontroller has an ultra-low-power 16-bit architecture that allows controlling the intelligent peripherals to extend the autonomy of the system. 

#### 2.1.4. Display Unit

Different low power solutions could be used to show the detection result, like printed and flexible electrochromic displays [49,50]. In this study, a 3-digit 7-segments numerical liquid crystal display (LCD) has been used to show the result, due to its simple operation and low power consumption, it also operates at 3 V with a typical quiescent current of 0.33 µA.

This kind of LCD uses many interconnects, to facilitate connection and control of the LCD, the CD4055B integrated circuit (IC) (Texas Instruments; Dallas, Texas, USA) has been used. It reduces the number of control signals needed to control the display and typically consumes 5 µA. This IC is a single-digit BCD-to-7-segment decoder that allows controlling the 7-segments of each digit with only four pins. The display is controlled by the display-frequency input signal, which is a square-wave signal. The IC provides a square-wave signal to the selected segments that is 180 degrees out-of-phase with the common-signal, making these segments visible. The segments which are not selected have the square-wave signal and are in phase with the common-signal, so they are not visible. The consumption of the DU module is 16 µA.

### 2.2. Fuel Cells and Sensors

**Figure 4:**
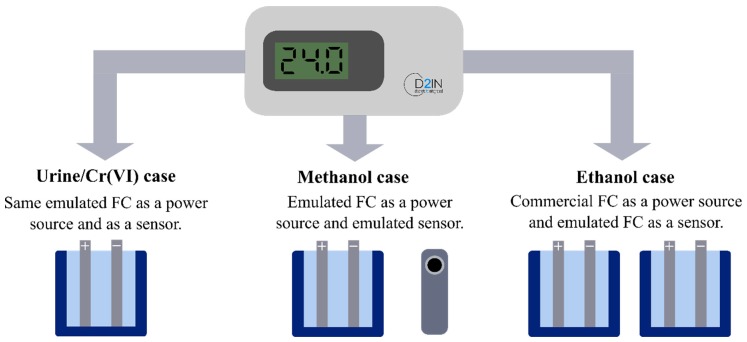
Summary diagram of the validation tests performed.

In order to validate the operation of the e-reader, we emulated different FCs and sensors reported in the literature(Figure 4). We emulated FCs and sensors based on urine/Cr(VI) [41] and methanol [51,52] by using a source measurement unit (SMU). Moreover, we carried out experimental tests with an emulated ethanol FC as a sensor [40] and a commercial ethanol FC as a power source.

These approaches fully validated the e-reader implementation. For each case, key design parameters are indicated, like the open-circuit voltage (OCV), and the related concentrations of the involved sample.

#### 2.2.1. Urine/Cr(VI) Fuel Cell Case

The urine/Cr(VI)’s FC presented by [41] is able to generate electrical power from processing human urine and heavy metal, in this case, Cr(VI). This FC reduces Cr(VI) in human urine, using urine as fuel and Cr(VI) as oxidant. The open-circuit voltage (OCV) ranges from 1.11 V at 13 mg·L^−1^ to 1.26 V at 50 mg·L^−1^. In addition, this FC provides a maximum power density going from 3.4 W·m^−2^ at 50 mg·L^−1^ of Cr(VI) to 2.2 W·m^−2^ at 13 mg·L^−1^ of Cr(VI). To validate the operation of the proposed system, we used the same urine/Cr (VI) FC as a sensor and as a power source.

#### 2.2.2. Methanol Fuel Cell and Sensor Case

In this case, we used a methanol FC as a power source and as a sensor to detect methanol. We emulated and employed a passive direct methanol fuel cell (DMFC) as a power source for portable electronic devices [51]. In the study, the authors used six dual DMFC connected in series to produce energy. In order to validate the e-reader operation, we considered a single FC of 1 cm^2^ with a 1 M of methanol concentration. It was only necessary to use a single FC because the developed e-reader was a low power device and operated with a minimum voltage of 330 mV. A single DMFC with a 1 M of methanol concentration provides a maximum power density of 5 mW·cm^−2^, a maximum current density of 22 mA·cm^−2^ and an OCV of 0.6 V. 

The emulated sensor is reported in [52]. It is a wearable vapor/liquid amphibious electrochemical sensor for monitoring methanol. The sensor exhibits high selectivity, good repeatability, and reliable stability for both vapor and liquid methanol. It was tested with methanol concentrations that go from 0% to 6%, providing current densities that go from 90 to 3.5 µA·mm^−2^. We validated the e-reader considering a methanol sensor of 1 mm^2.^

#### 2.2.3. Ethanol Fuel Cell Case

After emulating different approaches based on fuel cells and sensors, we carried on experimental verification working with a commercial FC. To perform the experimental test with the e-reader, we used as a power source the commercial FCJJ-42 ethanol fuel cell science kit (Horizon Fuel Cell Technologies; Singapore). Due to the fact that the characteristics of this FC are not described, we characterized it, obtaining the I–V curves for different ethanol concentrations that go from 3% to 9%. This ethanol FC provides a maximum power going from 0.23 W at 9% to 0.186 W at 3%. The maximum current given is 700 mA at 9% and 540 mA at 3%. All concentrations tested presented an OCV close to 1 V. To validate the system, we used the commercial ethanol FC with 3% of concentration as a power source.

The chosen FC used to sense ethanol was presented in [40]. It is a single-cell membraneless microfluidic FC that operates in the presence of simulated body fluids, human serum, and blood enriched with ethanol as the fuel. It provides current densities up to 6.5 mA·cm^−2^. 

### 2.3. Experimental Set-Up

The electronics validation and characterization were carried out with a source measurement unit (SMU) B2962A by Keysight Technology (Santa Rosa, CA, USA) and an Agilent Technologies oscilloscope MSO-X 3034A (Santa Clara, CA, USA).

In order to emulate the FCs and sensors previously presented, we introduced their I–V polarization curves into the SMU and performed a piecewise linear (PWL) interpolation. The PWL interpolation is a technique used in engineering to approximate a complex function by a simple linear function, which allows expressing the non-linear I–V characteristics that present FCs. Next, we analyzed the start-up of the power management unit (PMU) and emulated the current (I_CELL_) provided by the sensors and FCs.

Furthermore, we obtained the I–V curves in potentiodynamic mode at a scan rate of 2 mV·s^−1^ to characterize the ethanol FC. We performed the ethanol concentrations with deionized water obtained from a Milli-Q^®^ Advantage A10 water purification system and absolute ethanol for analysis (ACS grade) at a concentration of 99.8% (Panreac, Barcelona, Spain). 

## 3. Results and Discussion

### 3.1. Electronic Reader Manufacturing

**Figure 5:**
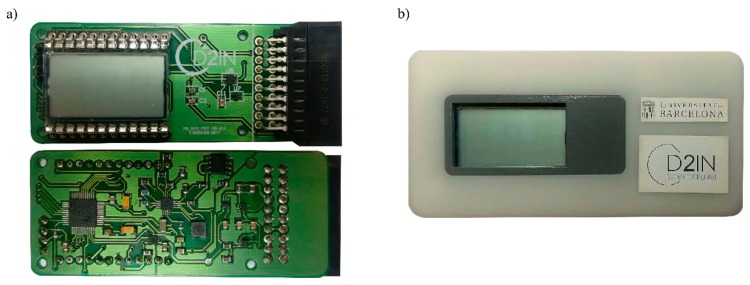
(**a**) Picture of the e-reader’s printed circuit board (PCB). (**b**) Picture of the whole self-powered electronic reader: PCB and outer case.

We developed a printed circuit board (PCB) and an outer case, both items build up the e-reader (Figure 5). The PCB is a double-sided printed circuit made in glass-reinforced epoxy laminate material (FR4) with silver-finish. The total size of the board was 77.5 mm × 32.5 mm × 2 mm. The case of the device had a size of 85 mm × 42 mm × 21 mm and was developed using three-dimensional (3D) printing. The case was made of a photosensitive epoxy resin called Accura^®^ 25 and it was created with the solid-state stereolithography (SLA^®^) printing technology.

### 3.2. Characterization and Calibration of the Self-Powered Electronic Reader 

We characterized the e-reader in order to analyze its performance and uncertainty translating the cell current (I_CELL_) into a voltage (V_OUT_). The procedure followed was previously explained, in which the SMU and the oscilloscope were used. 

**Figure 6:**
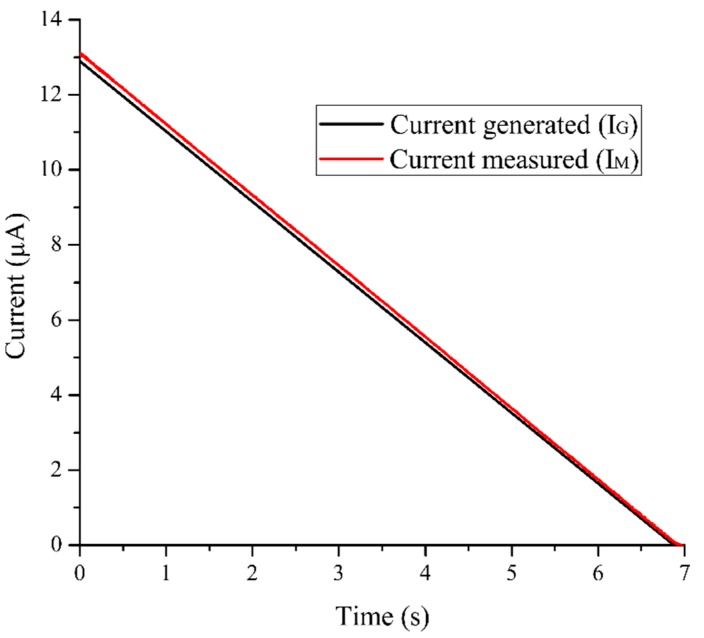
Uncertainty between the current generated by the SMU (I_G_) and the current measured by the front-end unit (I_M_).

First, we connected the SMU to the e-reader and we applied a current ramp (I_G_) from 13 µA to 0 µA with a change rate of 1.85 µA/s. Then, we captured the potentiostat output voltage (V_OUT_) by the oscilloscope. We translated V_OUT_ into the measurement current (I_M_) with Equation (1). As is shown in Figure 6, we compared the current measured by the e-reader (I_M_) with those applied by the SMU (I_G_). This test allowed us to obtain the uncertainty between the current generated (I_G_) and the current measured by the e-reader (I_M_). Analyzing these data, we obtained that the maximum relative uncertainty between I_G_ and I_M_ is 1.8%, which is the uncertainty associated with the FEU module. This maximum uncertainty was obtained at a nominal current value of 12.91 µA, and it had an absolute uncertainty value of 0.24 µA.

As it is stated in Section 2.1.3., the definition of the moment in which the measurement takes place, TM, is the key point. It depends mainly on two aspects: (a) The device resolution, which is constrained by the electronics’ resolution, and (b) the measurement uncertainty. This depends on the change rate of the current because a slight deviation in the measurement time can affect the current value, since initially the current decreased quickly. In order to obtain the ideal moment to perform the measurement, the e-reader was calibrated for each sensor. 

First, we established the polarization voltage (V_IN_) from the sensor’s characterization, and we programmed the e-reader to drive the sensor to V_IN_. Then, we connected the sensor to the e-reader and polarized it. After that, we captured the chronoamperometry curves with the SMU and analyzed the device resolution and the measurement. 

The e-reader resolution is defined by the ADC resolution, which is time-invariant and has a value of 13 nA. 

In a chronoamperometry, a redox reaction occurs when a potential step is applied to the electrode. The current decays t^1/2^, obeying the Cottrell equation for reactions that are under diffusion control [53]. Consequently, the initial and the final region cannot afford to extract a measurement. During the initial time, a small deviation in the moment to extract the measurement is translated into a large current uncertainty. While in the final region, it is difficult to distinguish between concentrations because they are too close. 

Considering these facts, we found the measurement uncertainty for different concentrations. As a result, we were able to establish the best moment to extract the measurement (TM). The time chosen in each case was the one which presented the lower uncertainty value for the worst case of detection, which was the detection of the lowest level of concentration. 

### 3.3. Start-Up and Power Consumption of the E-Reader

**Figure 7:**
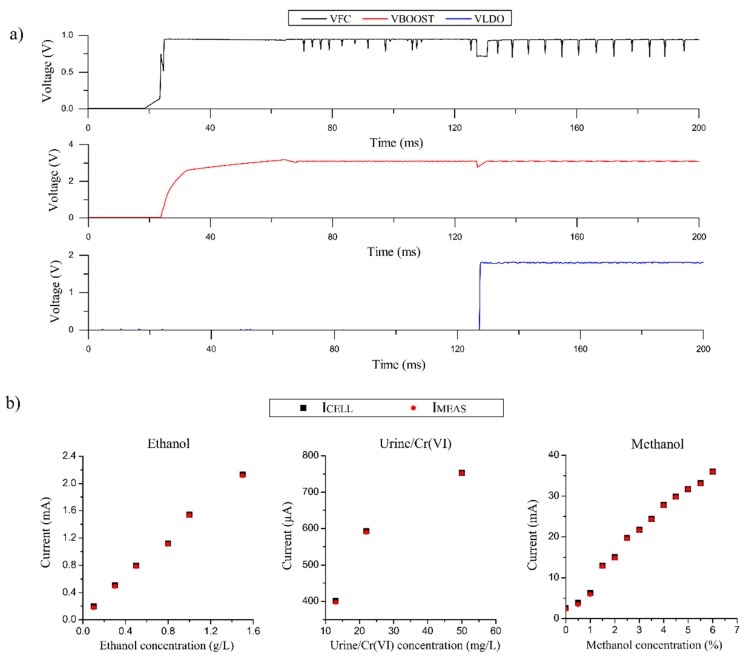
(**a**) Start-up curves of the e-reader powered by an ethanol FC. (**b**) Transfer function that relates the measured current by the e-reader with different emulated sensors and fuel cell concentrations (ethanol, urine and methanol), and the comparison against the current produced by the sensor (I_CELL_).

Figure 7a shows the startup curve graph of the e-reader when it is powered by a commercial ethanol FC as an example case. The upper graph shows the voltage provided by the ethanol FC (V_FC_). While, the middle graph shows the boost converter voltage (V_BOOST_), which is used to supply both CSPU and the DU. In the end, the bottom graph displays the voltage provided by the low-dropout voltage regulator (V_LDO_), which supplies the analog components that comprise the FEU. 

When the FC is connected to the e-reader, the PMU is enabled. It starts collecting energy and the V_BOOST_ signal increases. During 100 ms the V_LDO_ signal remains in a low state since there is not enough energy to start the whole system. When the PMU has harvested enough energy, the V_LDO_ signal changes to a high state and the system begins to operate. The peaks that appear in the V_FC_ signal correspond to the MPPT sampling system that optimizes the energy extraction from the power source. As it is shown in Figure 7a, V_FC_ is a noisy signal. For this reason, V_FC_ did not power any electronic components directly. The periodical peaks of V_FC_ were reduced by DC/DC boost converter (V_BOOST_), which supplies both CSPU and the DU. The FEU was the most sensitive circuit because it is the circuit that carried out the measurement. Thus, the voltage which supplied the FEU circuit (V_LDO_) was stabilized and regulated by a low-dropout voltage regulator (LDO).

We also obtained the e-reader’s power consumption. When the power source was connected to the e-reader, it had a typical inrush current peak of 5 mW. At this moment, the e-reader enters into a low power operation mode, and consumes only 900 µW. The system waits in this low power mode until the voltage provided by the power source is stable. Then, the FEU module is switched on and the measurement is performed. Lastly, the numerical data is processed and displayed, and the e-reader returns to the low power mode. During the device’s operation, the power consumption presents two peaks that increase the consumption instantly to 1 mW. These peaks occur when the FEU module is activated and when the data is processed and displayed.

### 3.4. Validation of the E-Reader

The system has been validated in different conditions and cases, with FCs of urine/Cr(VI), methanol and ethanol, and the methanol sensor. 

We verified the efficient extraction of energy from the sample in different cases, enabling the operation of both the DC/DC boost converter and the LDO, components that supply the FEU and the CPSU. The transitory performance of the commercial FC was obtained, extracting the curves shown in Figure 7a. This figure shows the behavior of the power signals in the “disconnected phase” and the “start-up phase”, explained in Figure 3. Moreover, we validated the stationary behavior of the device with the emulated FCs of methanol and urine. These tests confirmed the self-powered performance of the system, which can power the e-reader circuits with the same monitored sample.

Furthermore, we validated the FEU’s module operation, measuring different current ranges produced by the FCs of urine/Cr(VI) and ethanol, and the methanol sensor. For all these experiments, we followed the same procedure. Firstly, we connected the sensor to the e-reader, and we polarized it to the proper bias voltage (V_IN_). When the sensor is polarized, it produces a current proportional to the concentration of the analyte (I_CELL_). I_CELL_ flows through the potentiostat circuit, which translates it into the voltage (V_OUT_). The microcontroller’s ADC reads V_OUT_ and translates it to current (I_MEAS_) by a LUT, as stated in Section 2.1.3.

Figure 7b shows the comparison between the current produced by the emulated FC/sensor (I_CELL_) and the current measured by the e-reader (I_MEAS_). The figure depicts that the current measured by the e-reader is practically the same as the nominal current injected by the emulated FC. The results show that the maximum difference between I_CELL_ and I_MEAS_ is 1.8%, validating the operation of the FEU module of the e-reader with different ranges of current and demonstrating the accuracy of the e-reader.

These tests validated the operation of the system, demonstrating the ability of the device to power the whole system while measuring the concentration of the sample and displaying the numerical result on the e-reader’s display. The validation has been carried out with three different FCs, although it can be adapted to operate with other FCs, measuring a wide range of analytes.

## 4. Conclusions

In this work, we presented and described, in detail, the full implementation of an electronic reader for self-powered POC solutions based on the use of FCs as a power source or as a power source and as a sensor. The operation of the e-reader was validated in different cases, using a commercial ethanol FC, three emulated FCs (urine, ethanol, and methanol) and a sensor (methanol), showing its capability of adaptation to different scenarios. 

Due to its low power design, the platform can operate with a minimum voltage of 330 mV, consuming only 900 µW in the low power mode. The device has been proven to exhibit reliable, robust, and effective results. It has a measurement uncertainty below 1.8%, a minimum resolution of 13 nA and a maximum measurement current of 3 mA. Furthermore, the plug-and-play device performs amperometric measurements automatically, in only a few seconds. The device presented in this work could be the first step towards the future of point-of-care since the system provides quantitative data, showing the numerical result on the display. Some advantages of the proposed device include: (i) No need for external power sources, since the same sample is used to power and sense; (ii) the ease of use; (ii) compactness and portability; (iii) the possibility of showing the data on a display, without the need for external laboratory equipment; (iv) adaptability to wide range of cases; and (v) the rapidity and accuracy of results. The portable device can operate with conventional lithium batteries or biodegradable batteries [25] or with the sensing sample. It is a valuable characteristic in disadvantaged regions without a good electricity grid and batteries.

As future work, we propose to introduce a wireless communication system to show the resulting data on a smartphone or a laptop, a module to set the polarization voltage (VIN) externally, or an adjustable signal generator to perform cyclic voltammetry. A big challenge would be to integrate the device in a single chip, developing the system in a flexible substrate.
